# Modulating the Activity of the DLPFC and OFC Has Distinct Effects on Risk and Ambiguity Decision-Making: A tDCS Study

**DOI:** 10.3389/fpsyg.2017.01417

**Published:** 2017-08-22

**Authors:** Xiaolan Yang, Mei Gao, Jinchuan Shi, Hang Ye, Shu Chen

**Affiliations:** ^1^School of Business and Management, Shanghai International Studies University Shanghai, China; ^2^Academy of Financial Research, Zhejiang University Hangzhou, China; ^3^College of Economics, Zhejiang University Hangzhou, China; ^4^Neuro and Behavior EconLab, Zhejiang University of Finance and Economics Hangzhou, China; ^5^Interdisciplinary Center for Social Sciences, Zhejiang University Hangzhou, China

**Keywords:** risk decision-making, ambiguity decision-making, dorsolateral prefrontal cortex, orbital frontal cortex, transcranial direct current stimulation

## Abstract

Human beings are constantly exposed to two types of uncertainty situations, risk and ambiguity. Neuroscientific studies suggest that the dorsolateral prefrontal cortex (DLPFC) and the orbital frontal cortex (OFC) play significant roles in human decision making under uncertainty. We applied the transcranial direct current stimulation (tDCS) device to modulate the activity of participants’ DLPFC and OFC separately, comparing the causal relationships between people’s behaviors and the activity of the corresponding brain cortex when confronted with situations of risk and ambiguity. Our experiment employed a pre–post design and a risk/ambiguity decision-making task, from which we could calculate the preferences via an estimation model. We found evidences that modulating the activity of the DLPFC using right anodal/left cathodal tDCS significantly enhanced the participants’ preferences for risk, whereas modulating the activity of the OFC with right anodal/left cathodal tDCS significantly decreased the participants’ preferences for ambiguity. The reverse effects were also observed in the reversed tDCS treatments on the two areas. Our results suggest that decision-making processes under risk and ambiguity are complicated and may be encoded in two distinct circuits in our brains as the DLPFC primarily impacts decisions under risk whereas the OFC affects ambiguity.

## Introduction

Human beings are constantly exposed to uncertainty situations in which the careful weighing of possible outcomes is required to make decisions. In certain situations, the probabilities of possible outcomes can be determined, such as the gamble of coin tossing, whereas more generically, the probabilities are unknown, such as the chance of a sunny day. These two types of uncertainty situations are, respectively, called risk and ambiguity (Ellsberg, 1961; Curley and Yates, 1985). The behavioral differences of people facing risk and ambiguity have been demonstrated in various experimental economic studies (Ellsberg, 1961; Camerer and Weber, 1992). Individuals who are required to make decisions rely deeply on information regarding the probabilities of any possible consequences (Frisch and Baron, 1988; Fox and Tversky, 1995). Several studies suggest that individuals are more willing to bet on risky outcomes than on ambiguous ones for the same benefits (Ellsberg, 1961; Camerer and Weber, 1992). Explanations for this ambiguity aversion have included informed opponents (Kuhberger and Perner, 2003; Hsu et al., 2005) and comparative ignorance (Fox and Tversky, 1995). However, because the majority of experimental studies have focused on the risk situation and have seldom considered ambiguity, the relationship between risk and ambiguity behavior is still far from settled.

In fact, people’s attitudes toward risk and ambiguity may be distinct constructs. One recent study indicates that changes in ambiguity attitudes are age-related but those in risk attitudes are not (Blankenstein et al., 2016). Recent neuroscientific studies also find that the decision-making processes under risk and ambiguity are separately encoded in our brains (Hsu et al., 2005; Knoch et al., 2006). Neuroimaging studies have indicated the significant roles of the dorsolateral prefrontal cortex (DLPFC) and the orbital frontal cortex (OFC) in human decision making under uncertainty (Hsu et al., 2005; Kuhnen and Knutson, 2005; Huettel et al., 2006; Krain et al., 2006; Lin et al., 2014). Nevertheless, the results of these studies are various. Some studies find that the DLPFC is activated during risk decisions, whereas the OFC is more active in the situation of ambiguity than in the situation of risk (Bechara et al., 1996). PFC-lesioned participants tend to make riskier decisions, whereas OFC-lesioned participants are not sensitive to the degree of ambiguity and risk (Hsu et al., 2005). On the contrary, the meta-analysis by Krain et al. (2006) indicates that ambiguous decision-making is associated with activity in DLPFC while risky decision-making is associated with activity in OFC.

Because neuroimaging studies can hardly demonstrate a causal relationship between the activity of certain cortex areas and the specific types of preference, brain stimulation techniques are needed to investigate how modulating the cortex activity of the DLPFC and OFC will differently affect participants’ preferences for risk and ambiguity. Such studies find evidence that modulating activity in the DLPFC using transcranial magnetic stimulation (TMS) and transcranial direct current stimulation (tDCS) can change people’s decisions in uncertainty situations. However, the results of these studies also remain controversial. Certain studies find a positive relationship between the activity level of the right DLPFC and risk-taking behaviors (Ye et al., 2015a,b, 2016; Huang et al., 2017), while others find a negative or insignificant relationship (Knoch et al., 2006; Fecteau et al., 2007a,b). These varying results may be attributed to the confusion of the risk and ambiguity tasks applied in the studies, such as the Rogers’ Risk Task (Rogers et al., 1999), the Balloon Analogue Risk Task and the risk measurement table (Ye et al., 2015b). In addition, there is a lack of research investigating the effect of modulating activity in the OFC on participants’ preferences of risk and ambiguity.

In this study, we used a tDCS device to separately modulate the activity of participants’ DLPFC and OFC, comparing the causal relationships between people’s behaviors and the activity of the corresponding brain cortex when confronted with situations of risk and ambiguity. Our experiment employed a pre–post design and a risk/ambiguity decision-making task, from which we could separate the participants’ preferences for risk and ambiguity and calculate the preferences via an estimation model derived from Hsu et al. (2005). By comparing the participants’ preferences before and after the stimulation, we were able to identify whether the various stimulations had changed the participants’ risk and ambiguity attitudes. We aimed to find distinct effects of different stimulation treatments on different behaviors of risk and ambiguity.

## Materials and Methods

### Participants

A total of 72 students from Zhejiang University (42 males, 30 females; mean age 21.94 years, ranging from 18 to 28 years) participated in our experiment. Recruited by the campus BBS, the students had diverse majors including Arts, Science, Social Science, Engineering, Agriculture, Medicine, etc. All participants were right-handed and declared no history of psychiatric illness or neurological disorders, with no experience of tDCS or risk/ambiguity decision-making tasks. To participate in the experiment, they were required to provide written informed consent approved by the Zhejiang University ethics committee. The participants were randomly assigned to three stimulation montages (right anodal/left cathodal, left anodal/right cathodal and sham stimulation) with two types of target area (DLPFC and OFC); thus, there were six different treatments with 12 participants in each treatment. The experiment lasted approximately 1 h, and each participant received an average of 55.29 RMB yuan (approximately 7.95 United States dollars) for their participation. Although several participants reported slight itches during the experiment, no adverse side effects regarding pain on the scalp or headaches were reported after the experiment.

### Transcranial Direct Current Stimulation (tDCS)

Transcranial direct current stimulation (tDCS) was delivered by a battery-driven stimulator (multichannel non-invasive wireless tDCS neurostimulator, Starlab, Barcelona, Spain) via two saline-soaked surface sponge electrodes (35 cm^2^) fixed on the scalp of the participant with a rubber belt. The current had a constant intensity of 2 mA, delivered for 20 min with 30 s of ramp up and down. This montage would induce cortical excitability change in the target area without causing any physiological damage to the participants. The anodal electrode would enhance cortical excitability while the cathodal electrode would inhibit it (Nitsche and Paulus, 2000). For the sham stimulation, the current was delivered only for 30 s once it reached 2 mA. However, the participants treated this as the regular process of the stimulation and were unaware of their stimulation types, according to the questionnaire after the experiment. This method of sham stimulation has also been shown to be reliable by previous literature (Gandiga et al., 2006).

**FIGURE 1:**
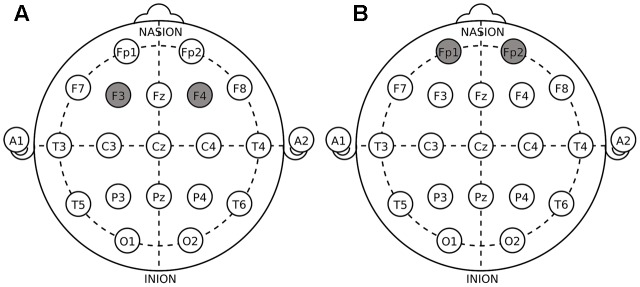
Electrode placements in dorsolateral prefrontal cortex (DLPFC) stimulations **(A)** and orbital frontal cortex (OFC) stimulations **(B)**.

**FIGURE 2:**
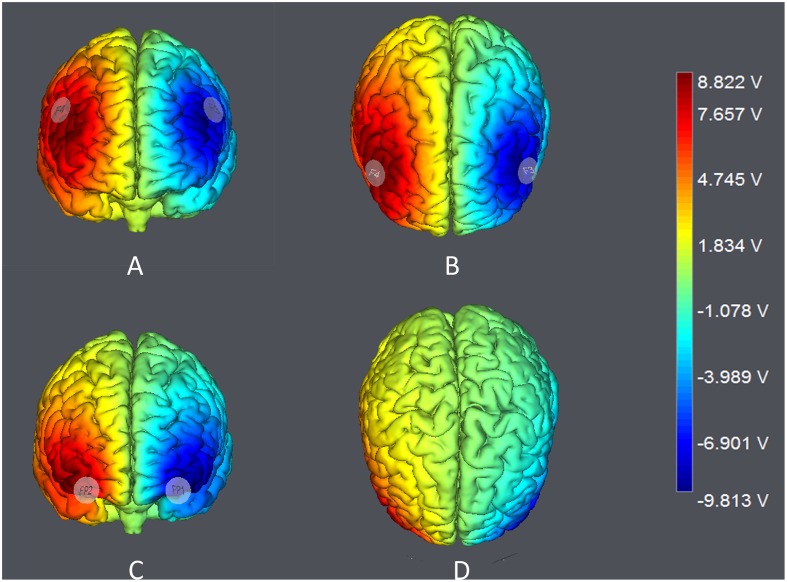
Electric simulations performed with the tDCS neurostimulator software. **(A,B)** simulations of right anodal/left cathodal DLPFC stimulation; **(C,D)** simulations of right anodal/left cathodal OFC stimulation.

The target areas were localized according to the International 10–20 System. For DLPFC stimulation, the anodal (cathodal) electrode was placed over the right F4 and the cathodal (anodal) electrode was placed over the left F3 in the right anodal/left cathodal (left anodal/right cathodal) treatment (**Figure 1A**). For OFC stimulation, the anodal (cathodal) electrode was placed over the right Fp2 and the cathodal (anodal) electrode was placed over the left Fp1 in the right anodal/left cathodal (left anodal/right cathodal) treatment (**Figure 1B**). These stimulation montages were proved effective in modulating the activity of DLPFC and OFC, respectively, by previous literatures (Fecteau et al., 2007a,b; Merzagora et al., 2010; Ouellet et al., 2015; Willis et al., 2015; Wang et al., 2016). Furthermore, **Figure 2** demonstrates the electric simulations of DLPFC and OFC stimulations performed with the tDCS neurostimulator software. We can see that the voltage distributions of the cortex were quite different between the two stimulation treatments. Electrodes placed over F4 and F3 mainly affected the DLPFC area while electrodes placed over Fp2 and Fp1 mainly affected the OFC area.

### Experiment Design

#### Risk/Ambiguity Decision-Making Task

The risk/ambiguity decision-making task is modified from Hsu et al. (2005), aiming to distinguish the participants’ preferences for pure risk and entire ambiguity via simple either-or choices. The task is composed of 48 choices; in each choice, the participants choose between a constant payoff (denoted as C.P.) and participation in a gamble. In the gamble, the computer will randomly select a ball from a given number of balls. There are only two types of balls, red and blue. In half of the choices, the composition of the balls is available to the participants, and thus, these choices will degenerate into pure risk decision-making (denoted as risk choices). In the rest of the choices, the composition of the balls is randomly decided by the computer and is unavailable to the participants, and thus, these choices will trigger ambiguity decision-making (denoted as ambiguity choices).

If the participant chooses the C.P., he (she) will obtain the exact amount of money. If he (she) chooses to take the gamble, then he (she) will obtain a higher gambling payoff (G.P.) when the red ball is selected but will obtain nothing if the blue ball is selected. The parameters of each choice are displayed in **Table 1**. For instance, if the participant chooses to take the gamble in Choice 1, the computer will randomly select a ball from 3 red balls and 1 blue ball. The participant will obtain 30 RMB yuan if it is red or nothing otherwise. If the participant chooses to take the gamble in Choice 48, the computer will first randomly decide the composition of the 40 balls and then select a random ball from them. The participant will obtain 25 RMB yuan or nothing depending on the result.

**Table 1:** The risk/ambiguity decision-making task.

	Risk choices				Ambiguity choices			
Choice Number	Total balls	Prob.	G.P.	C.P.	Choice Number	Total balls	G.P.	C.P.
1	4	3/4	30	21	25	1	28	14
2	4	3/4	17	13	26	2	22	7
3	6	5/6	30	20	27	4	22	9
4	6	5/6	23	20	28	6	24	9
5	6	5/6	26	17	29	7	28	16
6	6	5/6	21	14	30	7	28	15
7	7	5/7	19	15	31	9	28	10
8	10	9/10	27	18	32	10	29	17
9	12	5/6	22	17	33	15	24	11
10	15	2/3	23	13	34	15	26	15
11	15	2/3	26	16	35	15	28	15
12	15	2/3	17	11	36	15	23	9
13	15	2/3	17	11	37	19	30	17
14	15	2/3	20	10	38	20	17	6
15	16	5/8	27	16	39	20	27	8
16	18	5/9	29	14	40	25	26	8
17	22	10/11	18	14	41	25	17	9
18	22	10/11	23	18	42	30	27	12
19	23	15/23	16	10	43	30	22	12
20	25	3/5	16	7	44	34	20	11
21	25	3/5	17	11	45	35	28	15
22	30	2/3	23	13	46	38	23	8
23	30	2/3	25	12	47	40	18	9
24	35	4/7	23	11	48	40	25	10

*The prob. in risk choices means the proportion of red balls in the given number of balls*.

#### Procedure

The experiment began with the risk/ambiguity decision-making task run by the experimental software z-Tree (Fischbacher, 2007). The 48 choices were mixed and presented randomly one by one. After the task, the experimenter placed a tDCS device on the participant’s head for stimulation and told him (her) to calmly rest for 20 min. The tDCS device was stopped and taken away when the stimulation ended.

Next, the risk/ambiguity decision-making task was administered for a second time with different choice orders. However, the participants were not informed and demonstrated ignorance of the two tasks’ identity according to the questionnaire. All participants were asked to complete the questionnaire concerning personal information and experiment-related feelings before receiving their payments.

When the participants finished the questionnaire, the computer randomly selected one choice from the 48 choices in each task. The payments of the participants were the combination of a show-up fee and the payoffs in the selected choices according to their decisions and the randomness of the computer selections.

### Data Analysis

The participants’ selections in the risk/ambiguity decision-making task indicated their preferences for risk and ambiguity, from which we could determine certain depicting parameters. The model for parameter estimation is derived from Hsu et al. (2005). It assumes that participants have the following expected utility function: U(p, γ, x, ρ) = π(p, γ)u(x, ρ), in which u(x, ρ) is the possible utility and π(p, γ) is the subjective probability. This type of linear utility function is widely used in economics (Savage, 1954/1972; Hsu et al., 2005; Bouyssou and Marchant, 2011). u(x, ρ) =x^ρ^, where *x* is the possible payoff while ρ ≥ 0 is interpreted as the preference for pure risk: ρ > 1 indicates risk preferring, ρ < 1 indicates risk aversion, and ρ = 1 indicates risk neutral (Huettel et al., 2006). π(p, γ) =p^γ^, where *p* is the probability for the payoff while γ ≥ 0 is interpreted as the degree of ambiguity aversion: γ > 1 indicates ambiguity aversion, γ < 1 indicates ambiguity preferring, and γ = 1 indicates ambiguity neutral.

For C.P. options, p = 1. For gamble options in risky choices, *p* is the proportion of red balls in the given number of balls (i.e., the prob. in **Table 1**), and γ = 1 is constrained because the probabilities are given with no ambiguity. For gamble options in ambiguity choices, *p* is assumed to be 0.5 because the probabilities are randomly decided by the computer, while γ is left for estimation. In each choice, if the utility of the C.P. option is higher than the gamble option, the participant is predicted to select the C.P. option; otherwise, he/she is predicted to select the gamble option. We applied the Nelder–Mead simplex algorithm to find the value of ρ and γ that maximized the number of correct predictions (Huettel et al., 2006). Because the parameter estimates can be highly uncertain in one estimation, we repeated the estimations for 100 times and chose the one with the maximum number of correct predictions as the estimated value of ρ and γ. To test the robustness of our estimated values with respect to the participant’s selections, we also drew 1000 bootstrap samples and calculated the bootstrap errors of the estimated values. The estimations were performed using MATLAB software (MathWorks, Natick, MA, United States).

We used multiple types of ANOVAs to analyze the behavioral data and the estimated parameter values of the participants. We successively examined the number of C.P. selections, the parameter values of the participants and the effect of stimulation on different parameters in different treatments. We also examined the gender difference of the participants in our experiment. Multiple comparisons were adjusted by Bonferroni correction. The critical level of significance was set at *p* < 0.05. All statistical tests were performed using SPSS20 (SPSSInc., Chicago, IL, United States).

## Results

### C.P. Selections

**FIGURE 3:**
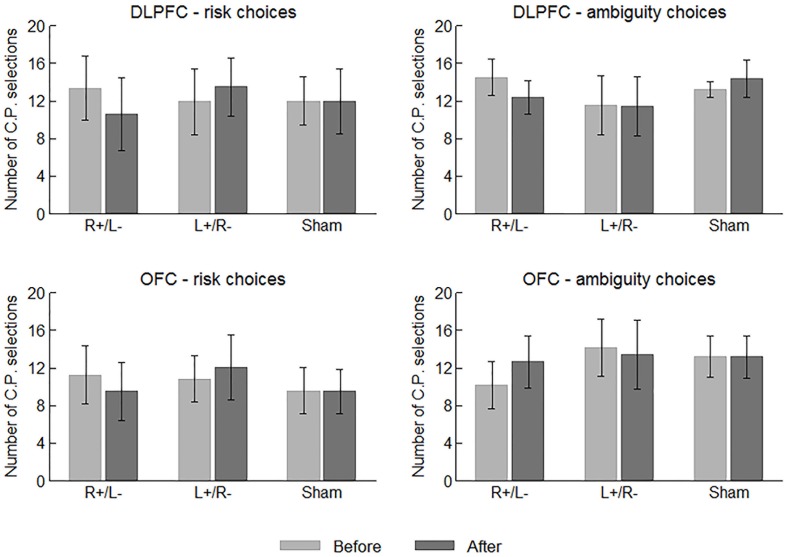
Average numbers of C.P. selections in different choices and treatments before and after the stimulations. Error bars indicate 95% confidence intervals.

We first examine the behavioral data of the participants. In each risk/ambiguity choice, the participants should choose between the C.P. and the gamble. More C.P. selections can roughly indicate a stronger tendency of risk/ambiguity aversion before we obtain the estimated parameter values. **Figure 3** displays the average number of C.P. selections in different choices and treatments before and after the stimulation. We performed one-way ANOVA using area (DLPFC vs. OFC) and stimulation type (right anodal/left cathodal, left anodal/right cathodal or sham) as the factor, respectively, while the number of C.P. selections served as the dependent variable. No significant effect of area or stimulation type was indicated on the number of both risk and ambiguity C.P. selections before the stimulations. In this sense, the participants were well mixed.

### Parameters

**FIGURE 4:**
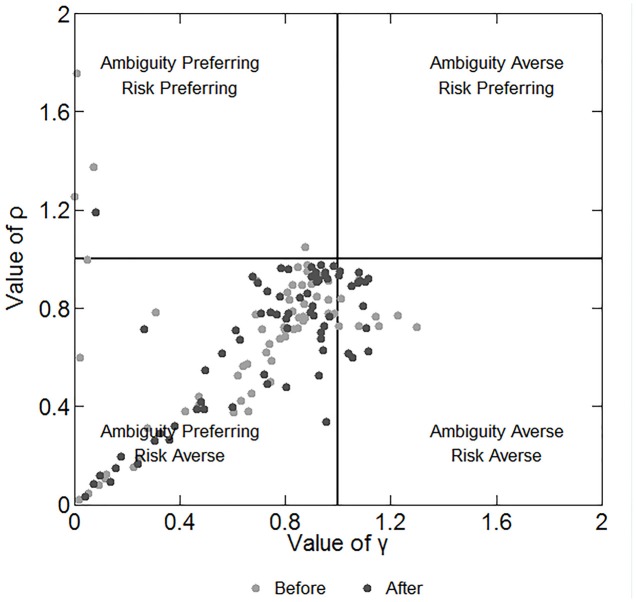
Estimated parameters and the corresponding preferences of participants before and after the stimulations. The *X*-axis depicts the degree of ambiguity aversion and the *Y*-axis depicts the degree of risk aversion.

We can infer the participants’ preferences for risk and ambiguity by the estimation model in subsection “Data Analysis.” This model is much more precise in depicting participants’ behaviors than the number of C.P. selections because it considers the different payoffs and probabilities of each choice. We repeated the estimations for 100 times and chose the one with the maximum number of correct predictions as the estimated value of ρ and γ. **Figure 4** shows the estimated parameters and the corresponding preferences of the participants before and after the stimulation. The plot region is divided into four quadrants, representing four combinations of risk and ambiguity preference. Each marker represents a single participant. We note that in **Figure 4** the majority of the participants are located near the middle of the plot region in the lower left quadrant, meaning that they are slightly ambiguity preferring and risk averse. However, some participants are much more ambiguity preferring and risk averse. Spearman’s rho test indicates a significant positive relationship between the values of γ and ρ (correlation coefficient: 0.490; *p* < 0.001). This finding reveals that the more ambiguity preferring the participant is, the more risk averse he/she is, and vice versa.

**FIGURE 5:**
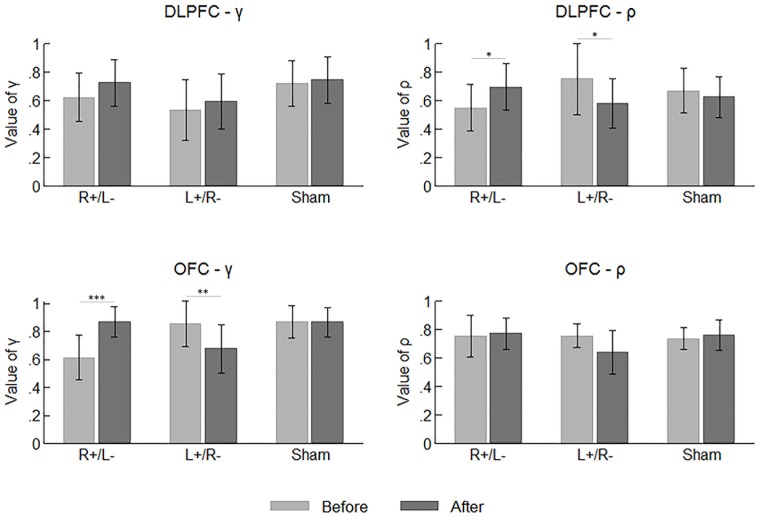
Mean values of the participants’ parameters before and after the stimulations. Error bars indicate 95% confidence intervals. Asterisks indicate statistical significance of difference.

**Figure 5** summarizes the mean values of the participants’ parameters before and after the stimulation. Again, we tested the difference across the area and stimulation type before the stimulations using one-way ANOVA with the parameter values as the dependent variable. Significant effect was observed on neither of the two parameters.

### Effect of tDCS

By comparing the parameter values before and after the stimulation, we can determine whether the tDCS changed the participants’ preferences for risk and ambiguity in different treatments. We applied a repeated measures ANOVA with parameter (γ vs. ρ) and time (before vs. after stimulation) as within-subject factors, while area and stimulation type served as between-subject factors. We found a significant effect of the interaction of time and stimulation type (*F*_2,66_ = 9.655, *p* < 0.001). The tests of simple main effect indicated that the parameter values were higher after the right anodal/left cathodal stimulation (before: mean = 0.635; after: mean = 0.766; *p* = 0.001) and lower after the left anodal/right cathodal stimulation (before: mean = 0.725; after: mean = 0.623; *p* = 0.009). We also found a significant effect of the interaction of time and parameter (*F*_1,66_ = 4.046, *p* = 0.048), indicating the value of γ was significantly larger than ρ after the stimulations (γ : mean = 0.746; ρ : mean = 0.681; *p* = 0.018). More importantly, there was a significant effect of the interaction of parameter, time, area and stimulation type (*F*_2,66_ = 6.468, *p* = 0.003). Simple main effect tests showed divergent effects of different treatments on different parameters. The value of γ was significantly improved by the right anodal/left cathodal tDCS on OFC (before: mean = 0.612; after: mean = 0.869; *p* < 0.001), whereas it was significantly decreased by the left anodal/right cathodal tDCS on the same area (before: mean = 0.857; after: mean = 0.678; *p* = 0.004). Conversely, the value of ρ was significantly improved by the right anodal/left cathodal tDCS on DLPFC (before: mean = 0.551; after: mean = 0.697; *p* = 0.048) but significantly decreased by the reversed tDCS on the same area (before: mean = 0.753; after: mean = 0.582; *p* = 0.021). In other words, the right anodal/left cathodal stimulation on the OFC made the participants more ambiguity averse and the same stimulation on the DLPFC made the participants more risk preferring, while the reversed tDCS on the two areas had the corresponding reversed effects. No significant changes in parameter values were observed in the sham stimulation.

### Gender Difference

Because the gender of the participants was not perfectly balanced in our experiment (42 males and 30 females), we further checked whether there was any gender difference in participants’ behaviors that may have influenced our results. One-way ANOVA showed no significant difference between males and females in the parameter of γ (*F*_1,70_ = 0.511, *p* = 0.477) or the parameter of ρ (*F*_1,70_ = 0.823, *p* = 0.367) before the stimulation. In addition, we added gender to the repeated measures ANOVA in subsection “Effect of tDCS”; again, no significant effect of gender or its interaction with other factors was observed.

### Bootstrap Errors

**FIGURE 6:**
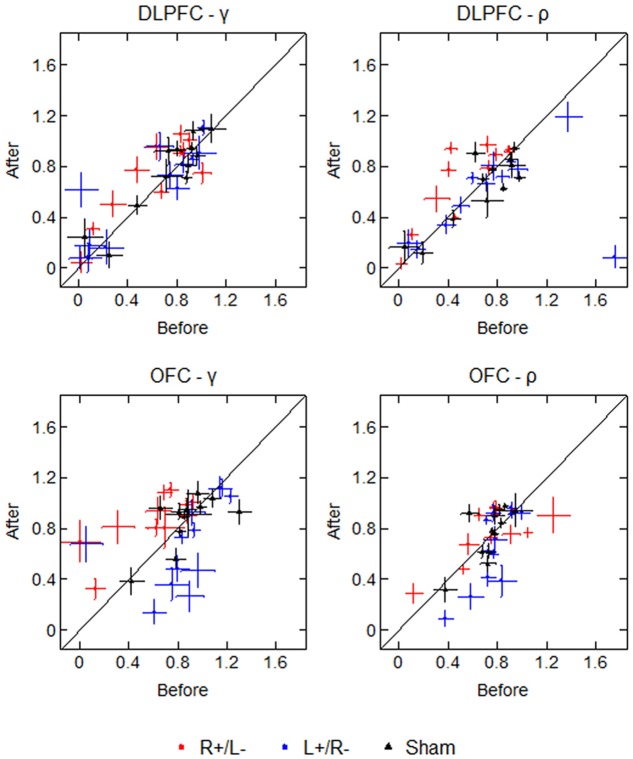
Parameter values and bootstrap errors before and after the stimulation in different treatments. Each marker represents a single participant’s parameter value, with crosses representing the bootstrap standard deviation.

To test the robustness of our estimated values with respect to the participant’s selections, we drew 1000 bootstrap samples and calculated the bootstrap errors of the estimated values for each participant. The average standard deviations of the bootstrap estimations of γ are 0.178 (before) and 0.174 (after), while the average standard deviations of the bootstrap estimations of ρ are 0.127 (before) and 0.120 (after). **Figure 6** displays the participants’ parameter values as well as the bootstrap errors of the 1000 bootstrap estimations. Each marker represents a single participant’s parameter value, with crosses representing the standard deviation of the 1000 bootstrap estimations. The diagonal line divides the plot region into two parts: the left part means that the mean parameter value was higher after the stimulation, and the right part means that the mean parameter value was lower after the stimulation. From the figure we can see that for some participants, the standard deviations of bootstrap estimations are non-trivial, which may weaken the robustness of our results.

## Discussion

This study used tDCS to investigate the effect of modulating the activity of the DLPFC and OFC on participants’ preferences for two types of uncertainty, namely risk and ambiguity. We applied a pre–post experiment design and a risk/ambiguity decision-making task from which we could calculate the participants’ preference parameters via an estimation model. We found evidence regarding the different neural basis of decision making under risk and ambiguity. Modulating the activity of the DLPFC using right anodal/left cathodal tDCS significantly enhanced the participants’ preferences for risk, while modulating the activity of the OFC with right anodal/left cathodal tDCS significantly decreased the participants’ preferences for ambiguity. The reversed effect was also observed in the reversed tDCS on the two areas.

The tDCS used in our study is a non-invasive brain stimulation technique that can modulate the activity of a certain area of brain cortex. Compared to brain imaging techniques, brain stimulation can better demonstrate the causal relationship between the activation of the target area and the corresponding behavior. Numerous neuroimaging studies have indicated a significant correlation between the DLPFC and risk decisions as well as the OFC and ambiguity decisions (Bechara et al., 1996; Hsu et al., 2005; Kuhnen and Knutson, 2005; Huettel et al., 2006; Lin et al., 2014). However, it is difficult to conclude whether the participants’ preferences for risk and ambiguity are attributed to the activity of the DLPFC/OFC, or whether there are other mechanisms involved in the decision-making process that may activate the corresponding areas. Using tDCS, we were able not only to enhance but also to inhibit the activity of the target areas to examine the causal effects of the DLPFC/OFC on risk and ambiguity preferences, which can help to better understand the neural mechanism of decision-making under uncertainty.

One of the crucial experiment designs of our study is the separation of risk and ambiguity. Previous experimental studies have indicated a behavioral difference in the participants confronted with risk and ambiguity situations (Ellsberg, 1961; Frisch and Baron, 1988; Camerer and Weber, 1992; Fox and Tversky, 1995; Blankenstein et al., 2016). Furthermore, neuroimaging studies have found a different neural basis of decision-making under these two types of uncertainty (Bechara et al., 1996; Hsu et al., 2005; Kuhnen and Knutson, 2005; Huettel et al., 2006; Knoch et al., 2006; Lin et al., 2014). However, the majority of brain stimulation studies use either risk tasks such as the Rogers’ Risk Task (Rogers et al., 1999) and the risk measurement table, or ambiguity tasks such as the Balloon Analogue Risk Task in which it is difficult to compare how the same stimulation will have different effects on the preferences for risk and ambiguity. Therefore, we applied the risk/ambiguity decision-making task to separate the participants’ preferences for risk and ambiguity. The task is modified from Hsu et al. (2005) and can delicately separate the two types of preference through the participants’ selections with an estimation model. We found significantly different effects of the DLPFC and OFC on participants’ risk and ambiguity attitudes: stimulation of the DLPFC is responsible for the attitude change in risk situations but not in ambiguity situations, while stimulation of the OFC is responsible for the attitude change in ambiguity situations but not in risk situations.

The close relationship between the DLPFC and risk behavior has been demonstrated by various brain imaging and brain stimulation studies (Bechara et al., 1996; Hsu et al., 2005; Kuhnen and Knutson, 2005; Huettel et al., 2006; Knoch et al., 2006; Fecteau et al., 2007a,b; Rao et al., 2008; Lin et al., 2014; Ye et al., 2015a,b, 2016; Huang et al., 2017). In our study, we found that enhancing the activity of the right DLPFC while inhibiting the activity of the left DLPFC significantly increased the participants’ preferences for risk, which is consistent with previous conclusions (Ye et al., 2015a,b). Meanwhile, we found that inhibiting the activity of the right DLPFC while enhancing the activity of the left DLPFC significantly decreased the participants’ preferences for risk. The right DLPFC has been indicated to be associated with cognitive regulation of emotions, especially the regulation of negative emotions and impulsive choice inhibition (Davidson et al., 2000; Ersche et al., 2005; Shackman et al., 2009; Schonberg et al., 2011; Yamamoto et al., 2015), and emotions are strongly involved in the risk decision process (Boggio et al., 2009; Mohr et al., 2010; Martin and Delgado, 2011; Peña-Gómez et al., 2011; Cohn et al., 2015; Engelmann et al., 2015). In this case, enhancing the right DLPFC may help to regulate or suppress the negative emotions such as the fear of failure and may inspire the participants to choose riskier behaviors.

Conversely, we found that enhancing the activity of the right OFC while inhibiting the activity of the left OFC significantly decreased the participants’ preferences for ambiguity, while the reversed montage had the reversed effect. Studies have found that the OFC is more active in situations of ambiguity than in situations of risk, and OFC-lesioned participants are not sensitive to the degrees of ambiguity and risk (Bechara et al., 1996; Hsu et al., 2005). The laterality in OFC is also reported as stronger effects are observed in the right OFC than in the left part during risk/ambiguity decision making (Hsu et al., 2005), and other studies found evidences of the lateralization of the activity within the right OFC (Milner, 1972; Tulving et al., 1994; Courtney et al., 1996; Haxby et al., 1996). Nevertheless, the laterality of OFC in risk/ambiguity decision-making remains seldom discussed. As an integration of the OFC in receiving emotional and cognitive input from the limbic system is implicated (Critchley et al., 2001; Hsu et al., 2005), our results indicate that the right OFC may play a more significant role in perceiving the negative emotions that occur during the ambiguity decision-making process, which inspires the participants to choose more ambiguity-averse behaviors. However, it is difficult to explain why the mechanism of emotion suppression of the DLPFC does not apply to ambiguity situations and why the mechanism of emotion receiving of the OFC does not apply to risk situations. Further investigations are needed to reveal the underlying neural difference between risk and ambiguity decision-making processes.

While the distinct effects of DLPFC and OFC on risk and ambiguity attitudes observed in our study is intriguing, it seems to be opposite to the meta-analysis by Krain et al. (2006) which reveals a significant association of DLPFC with ambiguity and an association of OFC with risk. One of the possible explanations for this inconsistency may be the difference in experimental paradigm. This study applies a risk/ambiguity decision-making task modified from Hsu et al. (2005) which can test the participants’ preferences for pure risk and entire ambiguity at the same time via simple either-or choices. However, the studies in the meta-analysis use several different tasks such as the Iowa Gambling Task, which is difficult to conclude whether it belongs to risk decision-making or ambiguity decision-making (Bechara and Damasio, 2005; Brand et al., 2007). In addition, some of the studies focus on unusual participants (e.g., methamphetamine dependent subjects and schizophrenia subjects) that may have neural differences compared to the participants in our study. Another concern is that the studies in the meta-analysis use either functional magnetic resonance imaging (fMRI) or positron emission tomography, which has quite different functional mechanism from tDCS. Krain et al. (2006) also mentions the limitations of fMRI in exploring median OFC’s activation as well as the potential bias resulting from the methodological limitations and the inclusion of the same investigator’s studies. All mentioned may lead to the inconsistent conclusions between Krain et al. (2006) and our study.

In our study, the majority of the participants were slightly ambiguity preferring and risk averse. Meanwhile, the more ambiguity preferring the participant is, the more risk averse he/she is, and vice versa. The participants’ risk preferences here are consistent with previous studies showing that people are risk averse when making decisions with positive outcomes (Kahneman and Tversky, 1979, 1984; Cohen et al., 1987; Smith et al., 2002; De Martino et al., 2006). Nevertheless, certain studies suggest that people are more willing to bet on risky outcomes than on ambiguous ones for the same benefits, which is known as ambiguity aversion (Ellsberg, 1961; Camerer and Weber, 1992). In addition, risk and ambiguity aversion are found to be positively correlated among investors (Butler et al., 2013). Our finding may be partly attributed to the different task and definition applied in our study because some studies displayed a reasonable impact of ambiguity type on attitudes (Chew et al., 2008, 2011). However, some researchers did find that participants mostly had ambiguity-neutral attitudes and seldom had ambiguity-averse or -preferring attitudes (Charness et al., 2013). Behavioral experiments in China have also found a wide range of attitudes, from ambiguity preferring to strong ambiguity aversion, among investors (Potamites and Zhang, 2012). In general, additional investigations are needed to verify people’s ambiguity attitudes and their relationship with risk attitudes, which could be a limitation of our study.

Another limitation would be the focality of tDCS. The technical constrains of tDCS mean that it is difficult to control the current flow and conclude whether the observed effects were due to selective modulation of the target area or rather due to the inevitable widespread and non-selective modulation over the cortex (Sellaro et al., 2016). The focality of our stimulation on DLPFC and OFC may be further limited by using the large electrodes and the bipolar scalp electrode arrangement (Gandiga et al., 2006; Nitsche et al., 2008). More convincing investigations would be those reducing the sizes of the electrodes to obtain more spatially limited excitability modification or applying an extracephalic return electrode as the reference (Accornero et al., 2007; Nitsche et al., 2007; Ferrucci et al., 2008). Furthermore, our bootstrap sampling indicated non-trivial standard deviations for some participants, which may weaken the robustness of our results. In this sense, our study provides some inspirations about the distinct roles of DLPFC and OFC on risk and ambiguity preferences, but still needs more robust evidences from studies in the future.

To some extent, our results suggest that decision-making processes under risk and ambiguity are very complicated and may be encoded in two distinct circuits in our brains; the DLPFC mainly affects decisions under risk, while the OFC affects decisions under ambiguity. Because decision-making under uncertainty is common in life, it is meaningful to explore its neural function to obtain a better understanding of people’s uncertainty decision behaviors and various cognitive diseases. Future studies may include the neural mechanism of the distinct effects of the DLPFC and OFC on risk and ambiguity decision-making processes as well as the behavioral relationship between the two types of uncertainty attitudes.

## Ethics Statement

This study was carried out in accordance with the recommendations of the Zhejiang University ethics committee with written informed consent from all subjects. All subjects gave written informed consent in accordance with the Declaration of Helsinki. The protocol was approved by the Zhejiang University ethics committee.

## Author Contributions

XY, MG, JS, HY, and SC designed the experiment. MG and SC performed the experiment. XY and SC analyzed the data. MG and HY drew the figures. XY, MG, JS, HY, and SC wrote the manuscript, revised the manuscript and finally approved the version to be published.

## Conflict of Interest Statement

The authors declare that the research was conducted in the absence of any commercial or financial relationships that could be construed as a potential conflict of interest.
